# A study protocol for *Truce*: a pragmatic controlled trial of a seven-week acceptance and commitment therapy program for young people who have a parent with cancer

**DOI:** 10.1186/s40359-015-0087-y

**Published:** 2015-09-09

**Authors:** Pandora Patterson, Fiona E J McDonald, Joseph Ciarrochi, Louise Hayes, Danielle Tracey, Claire E. Wakefield, Kate White

**Affiliations:** Research, Evaluation and Social Policy, CanTeen, Sydney, Australia; Institute of Positive Psychology and Education, Australian Catholic University, Sydney, Australia; Orygen, The National Centre of Excellence in Youth Mental Health, University of Melbourne, Melbourne, Australia; School of Education, University of Western Sydney, Sydney, Australia; School of Women’s and Children’s Health, UNSW Medicine, University of New South Wales, Sydney, Australia; Cancer Nursing Research Unit, Faculty Nursing, University of Sydney, Sydney, Australia; Behavioural Sciences Unit, Kids Cancer Centre, Sydney Children’s Hospital, Sydney, Australia

**Keywords:** Oncology, Parent, ACT, Intervention, Adolescent, Young adult, Cancer, Offspring, Well-being, Pragmatic trial

## Abstract

**Background:**

This paper presents the rationale and study protocol for a pragmatic controlled effectiveness trial of *Truce*, a prevention-based selective intervention targeting the significant mental health needs of young people who have a parent with cancer.

**Methods/Design:**

*Truce* is a seven week, facilitated, face-to-face group program. The design is a 2 groups (intervention vs control) x 3 (pre-treatment vs post-treatment vs 2 month follow-up) repeated measures. Allocation to groups will be dependent upon recruitment; when groups have sufficient numbers, they will be assigned to the intervention condition, but participants recruited without a viable group will be assigned to the wait-list control condition. Eligible participants are young people aged 14 to 22 years who have a parent diagnosed with cancer within the last 5 years. Wait-list controls are offered the opportunity to participate in the program once they have completed their follow-up questionnaires. The target sample size is 65 participants in each condition.

The primary hypothesis is that participants in the intervention will show significant reductions in distress and increases in psychological well-being relative to participants in the wait-list control group, and these effects will continue through two-month follow-up. Mixed-models analysis of variance will be used to measure differences between the two conditions. Secondary analyses will focus on variables which may relate to the effectiveness of the intervention: ACT-related concepts of experiential avoidance and mindfulness, family functioning, unmet needs and demographic variables. We will also assess program fidelity and satisfaction.

**Discussion:**

The development and evaluation of a manualised intervention for young people with a parent with cancer responds to a gap in the provision of empirically-based psychological support for this vulnerable group.

**Trial registration:**

Australian and New Zealand Clinical Trials Registry ACTRN12615000761561. Registered 22^nd^ July 2015.

## Background

### Supporting young people who have a parent with cancer

Each year, more than 21 000 Australian adolescents and young adults (aged 12–24 years) have a parent who is newly diagnosed with cancer (Patterson et al. 2014). For a young person, a parent’s cancer presents as a unique stressor with the threat of loss implicit in the diagnosis. Even when the prognosis is good, the young person may still be confronted by parental absences due to the need for hospitalisation, the ongoing impact of treatment side-effects upon the parent’s functioning, and disruption to normal family roles and routines, including shifts in responsibility for household duties and care of younger children onto the young people themselves (Romer et al. 2002; Welch et al. 1996; Maynard et al. 2013). Further, if the parent's cancer goes into remission, the young person still has to face the changes that the cancer brought, such as threat of recurrence and the possibility that the parent will no longer be able to act as carer (Osborn 2007). A young person’s experience of parental cancer is therefore likely to be disruptive and distressing across a number of psychosocial domains, with the added complication that the young person is already in a developmentally complex stage of life (Patton and Viner 2007).

Research on the psychological impact of having a parent with cancer has focused largely on young people’s internalising (e.g., anxiety and depression) and externalising (e.g., behavioural problems at school) symptoms as well as physical complaints (e.g., sleeping difficulties and headaches) and changes in cognitive function (e.g., maintaining concentration at school) (Visser et al. 2004). Research suggests that offspring of a parent with cancer exhibit relatively few externalising symptoms but that internalising symptoms are more common and elevated relative to community norms, especially among adolescents (as compared to young adults) (Visser et al. 2004; Osborn 2007). Furthermore, age and sex-related differences among offspring of parents with cancer generally indicate that adolescents and young adults experience more anxiety and depression than do pre-adolescent children, with the most elevated symptoms appearing in adolescent daughters of ill mothers (Compas et al. 1994; Romer et al. 2002; Osborn 2007; Welch et al. 1996; Visser et al. 2005).

The role of potential moderators of the psychological outcomes of young people who have a parent with cancer has also been examined. Key variables of interest have included: demographics (young person’s age and gender, parent’s gender, interaction between youth gender and parent gender); family make-up (parental marital status, presence/absence of siblings); disease variables (stage, prognosis, tumour type); treatment variables (no treatment, surgery, radiation, chemotherapy, combination of treatments); treatment outcomes/status (complications, side-effects, completion of treatment, recurrence of disease); time since diagnosis; prognosis; parent psychological functioning; family functioning (responsibilities, strain, roles, communication, cohesion, adaptability); and parenting (consistency, detachment) (Osborn 2007). Interestingly, disease and treatment variables do not appear to be associated with youth outcomes, nor do socio-demographic and parental mood variables, with the possible exception of maternal depression. The variables of greatest utility in predicting youth outcomes centre on family variables, especially those relating to family communication (McDonald et al. 2015; Osborn 2007).

Given the number of young people who have a parent with cancer, it is disheartening to note the scarcity of interventions specifically developed to assist this population (Lewis 2007; Romer et al. 2007). Recently, Niemela, Hakko and Rasanen (2010) conducted a systematic narrative review of interventions for offspring who have a parent with cancer and located only 11 structured child-centred interventions. They concluded that “..children are a forgotten group when families face a life-threatening parental illness.” (p.459). They found that interventions fell into two categories: structured family interventions and structured peer interventions. While results from family interventions were positive overall, the actual preventive effect of the interventions on children (for both the family and peer interventions) could not be evaluated due to the absence of follow-up. In general, the interventions lacked: a) methodological detail and theoretical underpinnings, b) reporting of results and use of valid outcome measures, c) substantial sample size, and d) control groups. All these factors prevent firm conclusions regarding the effectiveness of the interventions and highlight the pressing need for well-designed, controlled studies of interventions designed for the offspring of parents with cancer. The purpose of this paper is to provide researchers and practitioners with an outline of the development and content of a psychological intervention for young people who have a parent with cancer, and a rigorous study protocol designed specifically to evaluate the merits of an intervention.

### The Truce program

In consideration of the burden associated with a parent’s cancer diagnosis and the scarcity of programs available to support affected young people, there was a clear need for the development and evaluation of an intervention targeting the psychosocial and informational needs of this group. In response to this need, our research team^1^ developed *Truce*: a manualised 7-week, face-to-face group psychological intervention for young people who have a parent with cancer.

### Theoretical background – Acceptance and Commitment Therapy

*Truce* is part of an increasing movement to provide psychosocial support to people who present with risk, rather than waiting until a pathology is present, with the intention of reducing the likelihood of future psychosocial problems (Coie et al. 1993). This movement incorporates therapeutic paradigms that recognise client strengths and allow for growth (Kashdan and Ciarrochi 2013). Increasingly, the field of psychology has seen a shift from typical behavioural-only paradigms to cognitive - behavioural paradigms that emphasise changing the content of thoughts (Hollon and Beck 1994) to mindfulness-based therapies that emphasise changing one’s relationship with thoughts (e.g. Acceptance and Commitment Therapy, ACT). ACT teaches people to let go of destructive attempts to control feelings and to allow unhelpful thoughts to occur without “hooking to them” or believing them (Hayes et al. 1999). Overarching the ACT approach is the broader goal of increasing psychological flexibility, or the ability to adapt to shifting demands and maintain balance through the use of mental resources (Kashdan and Rottenberg 2010). In addition, ACT draws on the field of positive psychology, where the goal is to begin “… to catalyse a change in focus of psychology from preoccupation only with repairing the worst things in life to also building positive qualities” (Hayes et al. 1999, p. 5).

There are six core principles of ACT: *acceptance, defusion, being present, self as context, valued living and commitment to action* (Hayes et al. 1999; Bach and Moran 2008). *Acceptance* involves opening up in the service of values, and accepting painful internal experiences such as feelings and emotions instead of fighting them. *Defusion* refers to “unhooking” from thoughts; learning to observe them and not react to them, nor treat them as always true. *Being present* means consciously connecting to, and engaging with, the situation the person is currently in. *Self as context* refers to being aware of an internal, “observing self” distinct from the “thinking self”. *Valued living* involves identifying an individual’s personal values, that is, what is important to them. Finally, *commitment to action* refers to taking effective actions in a way that is consistent with one’s values (Hayes et al. 1999; Harris 2009).

### Previous ACT studies

Empirical evidence for the efficacy of ACT therapeutic approaches has previously been reviewed (Ruiz 2010). The review examined three types of study; namely, (i) correlational studies, (ii) experimental psychopathology and ACT component studies, and (iii) outcome studies. Results from the review highlighted a wide variety of conditions that have shown positive long-term outcomes from ACT treatment, including: depression, anxiety disorders, psychosis, chronic pain, and substance use. ACT therapies have demonstrated efficacy, producing large effect sizes that are typically maintained or increased at follow-up. Murrell and Scherbarth (2006) reported empirical support for ACT interventions across a variety of child and adolescent populations: adolescents at risk of dropping out of school (Moore et al. 2003), paediatric pain patients (McCracken et al. 2010), and adolescent girls either engaging in high-risk sexual behaviour (Metzler et al. 2000) or experiencing anorexia (Heffner et al. 2002). Further, research shows that for adolescents in school, ACT has good long-term effects at 2-year follow-up (Livheim 2004). Two randomised controlled trials for adolescents with depression provide further evidence of treatment efficacy (Hayes et al. 2011), and further recent evidence has been found for the effectiveness of ACT in helping adolescents with chronic pain (Gauntlett-Gilbert et al. 2012) and obsessive compulsive disorder (Armstrong et al. 2013). Finally, longitudinal research suggests that awareness and acceptance, both core concepts of ACT, are critical components of positive adolescent development (Ciarrochi et al. 2008, 2011; Marshall et al. 2015; Rowsell et al. 2014).

### The Truce facilitator manual, participant workbook and parent booklet

The working party involved in the development of *Truce* included consumer representatives who were young people impacted by parental cancer and psychologists with expertise in: developmental educational concerns, adolescent development, ACT, clinical work and young people impacted by cancer. A cancer nurse with clinical and research expertise and CanTeen staff with expertise in researching and working with young people and their families living with cancer, also contributed to the project.

The *Truce* program was developed to include all of the components of ACT (Hayes et al. 1999) and to gradually build and reinforce ACT skills and knowledge. It is a manualised, seven-session program (see Table 1) with the sixth session involving both the young person and their parent. In addition to the facilitator manual, a participant workbook and a parent’s booklet have been developed. The facilitator manual contains background information regarding the development and running of *Truce*, detailed content of each session, and all necessary worksheets and tools. The participant workbook contains review material for each session along with home practice activities which participants complete between sessions. Participants are given their workbooks in the first session and are not required to bring them to following sessions.

**Table 1:** Content of the seven *Truce* sessions

Session	Content
*Session 1*. What happened to my ‘normal’ life!?	Welcome, establishing program engagement and group guidelines. Introduction to ACT and overview of the program. Psycho-education about the changes and accompanying psychosocial difficulties associated with having a parent with cancer. Opportunity to talk about how having a parent diagnosed with cancer has changed their life. Assisting the group to process how their experiences are similar and different between participants; normalising feelings, fears and concerns. Introduction to dealing with feelings, and grief and loss.
*Session 2*. Dealing with feelings	Developing understanding about the futility of trying to control unwanted feelings. Learning to have a different type of relationship with feelings; being willing to acknowledge them and allowing them to be there.
*Session 3*. Being Mindful	Introduction to the concept of mindfulness; learning to notice thoughts, feelings and physical body sensations; and understanding the importance of connecting with the present moment. Practising mindfulness in everyday life. Introduction and practice of the BOLD acronym: Breathing, Observing, Listening and Deciding.
*Session 4*. Sticky thoughts	Explores the concept of wanted and unwanted thoughts and the futility of trying to control thoughts. Introduction to the idea of the mind as a problem-finding and problem-solving machine. Introduction to the concept of sticky thoughts and learning to have a different, healthier relationship with them.
*Session 5*. Getting unstuck	Teaches strategies for getting unstuck from difficult thoughts to be able to refocus energy on better listening to what is important to us (values) and taking committed action towards those values.
*Session 6.* Values and Loving - Kindness	A combined session that brings the parent and child together to work on communication, values and loving - kindness. Increasing understanding of the importance of values to how we live our lives and that staying stuck to difficult thoughts prevents us from living well and heading in the direction we want to go in life. Learning to be unstuck to be free to listen to our values and decide on our actions. Practising loving-kindness toward ourselves and self-compassion; and using “wise view” to accomplish this.
*Session 7*. Taking action!	Identifying values and goals, and committing to take action to live in line with them by developing action plans. An opportunity to review and reflect on what has been learned in the *Truce* program over the seven sessions.

Given the aforementioned importance of family variables, the *Truce* program has been developed with consideration of Olson’s Circumplex Model, a family-focused framework (Olson 2000). Parents or caregivers who choose to participate are involved in the evaluation questionnaires and attend session six. A parent does not need to participate in the program in order for his or her child to participate. Likewise, the parent with cancer does not necessarily have to be the parent that attends session six.

The Parent Booklet will be given to parents prior to the commencement of the *Truce* program. The purpose of this booklet is to provide parents with information about the impact of parental cancer on young people and how they can support their child. Parents are encouraged to read the booklet prior to the commencement of their child’s participation in the *Truce* program. The booklet contains information on the *Truce* program and home practice activities, to be completed by the parent after he or she has attended the joint session. Specifically, the parent booklet contains information from research on the needs of young people who have a parent with cancer (Patterson et al. 2011) and information about what is helpful for young people who have a parent with cancer (Maynard et al. 2013). The ‘How you can help your child?’ section of the booklet has been structured on Olson’s Circumplex Model (Olson 2000).

## Methods/Design

### Study aims

The primary aim of the study is to evaluate the effectiveness of *Truce*, a 7-week ACT-based program for young people (aged 14 to 22 years) who have a parent diagnosed with cancer.

The primary outcome variables are distress and psychosocial well-being of intervention-group participants, relative to control-group participants, across the duration of the study (i.e., from pre-treatment through post-treatment and two - month follow-up assessment).

### Hypotheses

It is hypothesised that greater reductions in distress and improvements in psychosocial well-being will be observed in *Truce* program participants than in wait-list controls. Secondly, these effects will be maintained at two-month follow-up.

### Study design and setting

The study utilises a 2 (intervention vs control) x 3 (pre-treatment vs post-treatment vs two-month follow-up) repeated measures, within-between participants design. The two-month follow-up time was selected to provide a measure of the sustainability of the *Truce* program post-intervention, while minimising the waiting time before wait-list control participants commenced treatment.

The CONSORT guidelines for pragmatic trials (Zwarenstein et al. 2008) were used in the development of the study protocol, with the exception of randomisation of participants to intervention conditions. Pragmatic trials differ in emphasis from other randomised controlled trials in that they focus on examining effectiveness (i.e., they attempt to maximise the applicability of the trial to usual care settings) rather than efficacy (i.e., examining the impact of the intervention under ideal trial conditions) (Zwarenstein et al. 2008). Accordingly, we aimed to maintain methodological rigour within the constraints of working within realistic settings. Assignment of participants to the intervention or wait-list control condition will be determined by whether there are enough participants in an area to run a group (four to eight is the acceptable number for a group); where there are not enough participants, they will be assigned to the wait-list control condition. While not a true randomisation of participants, this approach lends external validity as it is similar to the circumstances around administering group therapy programs in the community.

The *Truce* program will be offered in a number of locations throughout Australia. The group meetings are to be hosted in private rooms in a range of locations such as CanTeen offices, Cancer Council offices and public spaces such as libraries. The program will be facilitated by both CanTeen staff and external facilitators, all of whom have been trained in ACT methods and who are experienced at working with young people. In addition to ensuring facilitators are appropriately trained and qualified, the *Truce* program is manualised (as described above) and all materials will be provided. Weekly briefing and debriefing sessions will be held with all facilitators to ensure the program is delivered according to the manual.

### Participants

#### Inclusion criteria

Young people will be eligible to participate if they are: (i) able to provide informed consent; (ii) able to read English; (iii) aged between 14 and 22 years; and, (iv) have a living parent (or caregiver) who has been diagnosed with cancer within the last 5 years. Participating parents (and caregivers) are required to: (i) be able to provide informed consent; and, (ii) have a child involved in the intervention with whom they have an on-going relationship. Parent/caregiver participants need not be the biological parent of the young person, and can either be the diagnosed adult or a spouse.

#### Exclusion criteria

Young people are ineligible to participate if: (i) they have been bereaved; or (ii) they are identified as being highly distressed or in need of urgent support through either the screening phone call or their questionnaire responses.

### Sample size calculations

The sample will include a total of 130 young people who have a parent with cancer and, where possible, one parent of each young person. The number of required participants is based on sample size analyses assuming: 80 % power, alpha of 0.05 and a minimally important change in K10 scores of 4.9 (effect size of 0.5). According to these calculations, 65 participants are needed in each of the intervention conditions.

### Promotion and recruitment

Recruitment will take place through CanTeen, partner cancer support agencies, hospitals, youth organisations and schools. To maximise participant recruitment, a business marketing approach will be followed; the *Truce* brand will be marketed and project champions will promote the study (McDonald et al. 2011). A section of the existing CanTeen website dedicated to the project has been established (www.truce.org.au). This provides information on *Truce*, the study, as well as a contact form, links to participant information and consent forms, and information about study enrolment. The creation of the website was considered necessary to raise the profile of the *Truce* program and to improve engagement with potential participants. As it is not possible to estimate the number of eligible participants who will receive information on the *Truce* program, it will also not be possible to calculate response rates. Further details on the promotion and recruitment of *Truce* are discussed in elsewhere (McDonald et al. 2015).

### Consent, enrolment and screening

Potential participants may contact the research team directly or be referred to the *Truce* program. Initial screening will take place via a telephone call which will determine whether the young person is eligible to participate in the study and that he or she understands the research component of the program, including the possibility of being allocated to the wait-list control condition. Interested eligible participants will be mailed a participant information sheet and consent form. Under-age participants (i.e., those 17 years or younger) will require parental consent. A separate participant information sheet and consent form will be mailed to the parent if he or she is willing to take part in the study. Once the consent forms are completed and returned, the participant will be enrolled into the study and sent the baseline (pre-treatment) questionnaires.

### Ethics

This study received ethics approval from the University of Wollongong Human Research Ethics Committee, HE11-482. The study will be carried out in compliance with the WMA Declaration of Helsinki – Ethical Principles for Medical Research Involving Human Subjects.

### Wait-list control

Wait-list control participants will be provided with standard treatment which includes being sent a copy of CanTeen’s book, *Now What..? My parent has cancer* (CanTeen 2008)*.* They will also be offered the opportunity to participate in the *Truce* program after they have completed all wait-list data collection requirements. Wait-list control participants are not prevented from accessing other support, including other CanTeen programs, consistent with a treatment-as-usual approach.

### Measures

A range of measures will be administered throughout the *Truce* program. The timing of the measures is shown in Table 2. All measures except for fidelity measures (described below) can be completed on paper or online.

**Table 2:** Timing of measures used in *Truce* evaluation

Measure	T0	T1	T2	Each session
Demographics (Young people and parents)	X			
Young people: K10, RADS-2, Keyes, Brief COPE, FRI, OCNI, CAMM, AFQ-Y	X	X	X	
Parents: K10, FRI	X	X	X	
Satisfaction^a^		X		
Fidelity				X

^a^Satisfaction with *Truce* is measured for all young people and their parents who participate in the *Truce* groups at the completion of the *Truce* program regardless of whether they are in the intervention or wait-list control condition

### Outcome measures

#### Kessler10 (K10; Kessler et al. 2002)

Ten items measuring anxiety and depression symptoms experienced over the past four weeks. The scale has been used widely in studies with Australian adolescents and adults, including by the Australian Bureau of Statistics (2012). Responses are given using a 5-point Likert scale (None of the time to All of the time). The K10 has excellent internal consistency (α = 0.93; Kessler et al. 2002). All items use the sentence stem: *In the past 4 weeks how often did you feel*... An example item is: *tired out for no good reason?*

#### Reynolds Adolescent Depression Scale-2 (RADS; Reynolds and Mazza 1998)

Thirty items measuring current symptoms of depression in adolescents. Responses are given using a 4-point Likert scale (Almost never to Most of the time). An example item is: *I feel like running away*. The RADS has been shown to have excellent internal consistency (α = 0.93) and validity with other measures of depression and structured diagnostic interviews.

#### Keyes Psychological Well-being Scale (Keyes 2006)

Twelve items measuring: emotional well-being (3 items, e.g., *How often did you feel happy?*), psychological well-being (4 items, e.g., *Howoften did you feel confident to think or express your own ideas and opinions*?”), and social well-being (5 items, e.g., *How often did you feel that you belonged to a community like a social group, your school, or your neighbourhood*?). Responses are made using a 6-point Likert scale as to how often the item occurred for the respondent in the past month ("*Never*" to "*Every day*"). The measure has been shown to have high internal consistency across all three subscales (α = 0.78 to 0.84).

Brief COPE scale (Carver 1997) A 28-item scale measuring coping strategies. Respondents answer how often they have been using a strategy by selecting a response on a four-point Likert scale (*I haven’t been doing this at all*, to *I’ve been doing this a lot*). For this study, we will be using the following six subscales (total of 12 items) – Active Coping (e.g.,*I’ve been taking action to try and make the situation better*), Emotional Support (e..g.,*I’ve been getting emotional support from others*), Instrumental Support (e..g.,*I’ve been getting help and advice from other people*), Positive Reframing (e..g.,*I’ve been looking for something good in what is happening*), Planning (e.g.,*I’ve been thinking hard about what steps to take*) and Acceptance (e.g.,*I’ve been learning to live with it*). Internal consistency scores for the factors range from .57 to .74 and are all above .50 (the minimally acceptable level).

### Demographic measures

Variables include date of birth, sex, level of education, employment status, family structure, sex of parent with cancer, type of cancer, time since diagnosis and treatment stage.

### Measures for secondary analysis

Secondary analyses will determine potential moderators and mediators of the intervention. Moderators will help to answer the question, “For which individuals is the intervention most likely to work?” (MacKinnon 2011). These variables could impact on the effects of the *Truce* program on primary outcomes. Mediator measures will be useful in answering the question “How does the intervention work?” (MacKinnon 2011). Potential mediators are therapeutic processes that are components of ACT expected to be essential in facilitating improvements in the outcome measures. The following measures will be administered for the purpose of secondary analysis:

#### Offspring Cancer Needs Instrument (OCNI; Patterson et al. 2013)

Forty-seven items measuring levels of unmet need for young people who have a parent with cancer. Items are clustered into seven domains: Information about my parent’s cancer; Family issues; Practical assistance; ‘Time out’ and recreation; Dealing with feelings; Support from my friends; and Support from other young people. Items are answered according to the sentence stem: *I currently need*…, and a 4-point Likert scale:(*I don’t have any need for help with this issue* to*I have a strong need for help with this issue*). The OCNI has good psychometric properties with factor internal consistencies ranging from 0.89 to 0.96, and an overall test-retest reliability of 0.73.

#### Family Relationship Index (FRI; Moos and Moos 2009)

Twelve items from the Family Environment Scale covering three subscales: cohesiveness (e.g.,*There is a feeling of togetherness in our family*), expressiveness (e.g.,*We tell each other about our personal problems*) and conflict (e.g.,*We fight a lot in our family*). Respondents select true or false for each item based on the circumstances of their family. For this research both parents and young people will complete this measure. The internal consistency coefficients of the three subscales are 0.78, 0.69 and 0.75.

#### Child Acceptance and Mindfulness Measure (CAMM; Greco et al. 2011)

The short form of the CAMM has 10 items measuring mindfulness that assess the degree to which children and adolescents observe internal experiences, act with awareness, and accept internal experiences without judging them. Responses are made using a 5-point Likert scale (*Never true *to *Always true*). An example item is: *I tend to go from place to place without noticing what I am doing*. The CAMM has moderate to good convergent validity (*r* = 0.37-0.60) with measures of similar constructs.

#### Avoidance and Fusion Questionnaire for Youth (AFQ-Y8; Greco et al. 2008)

This instrument contains 8 items measuring psychological inflexibility engendered by cognitive fusion, experiential avoidance, and behavioural ineffectiveness in the presence of negatively evaluated private events (e.g., thoughts, feelings, physical sensations). Responses are made using a 4-point Likert scale (*Not at all true *to *Very true*). An example item is: *I can't be a good friend when I feel upset*. The AFQ-Y has good internal consistency (α = 0.90).

### Satisfaction measures

Satisfaction data will be collected from intervention group participants and parents at the second time point. Intervention group measures include questions on whether *Truce* helped participants to deal with their situation and whether they would recommend the program to someone else. There are also some open-ended questions about what participants liked or disliked about the program. The parents’ questionnaire contains similar items as well as questions on whether they have noticed any changes in their children.

### Fidelity measures

Program fidelity refers to adherence to the guidelines for delivering the program with consistency across facilitators and locations. Integrity to the manual will be addressed by holding weekly briefing and debriefing sessions for all facilitators throughout the program and through facilitators completing a post-session checklist detailing delivery of each session activity. It is also a requirement that all facilitators have training in ACT and experience working with young people. Participant attendance (“dose”) will be measured through an attendance checklist, supplemented by the facilitator recording that all components of a session were completed. Participant responsiveness, which is a record of how involved participants are with the program, will be measured using an “interest-o-meter” which has a scale from 0–10 to record how interesting participants find each session, and a homework check sheet which will record participant engagement with their homework.

### Program safety

Participants’ well-being will be monitored from initial contact through to program completion and follow-up. In addition to formal measures and the initial screening telephone call, facilitators will report on concerns that arise during their weekly debriefing sessions.

### Data management and planned statistical analysis

Descriptive statistics will be used to report demographic information. Fidelity and satisfaction measures will also be summarised. Missing data will be managed using multiple imputation techniques. The impact of the treatment condition on the change in outcome variables will be assessed using mixed-models statistical analysis. Secondary analyses (such as testing for potential moderators and mediators) will be conducted using multiple regression statistical techniques.

## Discussion

Young people who have a parent with cancer have many unmet needs (Compas et al. 1994; Patterson et al. 2011), but very little in the way of evidence-based programs that are designed to address these needs. Similarly, healthcare professionals who recognise the needs of these young people have few avenues for referral available to them. We hypothesise that *Truce* has the potential to provide considerable benefit to these young people, and that rigorous evaluation of the program will inspire healthcare professionals to refer to the program with confidence.

*Truce* has three clear strengths as a program for young people impacted by parental cancer. The first of these is that it is based on ACT, which has a considerable body of empirical evidence supporting its efficacy. ACT seems particularly suited to this client group for several reasons. ACT teaches young people to accept what can’t be changed, such as the threat of cancer, loss and the difficult feelings that arise. It also helps young people to engage in valued action, even in the presence of difficult feelings. Finally, ACT minimises the use of argument and complex problem solving and reasoning processes, which may particularly suit younger people whose abstract cognitive skills are still developing.

The second strength of the program is that it involves peer-to-peer interaction, which has been found to be an unmet need of young people who have a parent with cancer (Patterson et al. 2011). Participants are able to take part in a group where everyone is connected by a life circumstance that is often difficult to discuss on an everyday level with peers. Thirdly, it acknowledges the importance of a family systems approach.

The measures employed, as well as the use of a wait-list control group, will enable the impact of the program on the key outcome measures to be assessed. The longitudinal design will indicate whether post-intervention gains are maintained at follow-up. The design further allows us to identify factors that are likely to weaken or strengthen the effect of the intervention and the possible ACT processes behind the functioning of the intervention.

Recruitment is known to involve substantial challenges with studies of this type due to barriers in identifying young peoples' need for help. Raising awareness of the program is also challenging. A longer discussion of the challenges of recruitment for this study is to be presented in future publications. There is also limited potential to analyse the impact of the different elements of the program upon participant outcomes due to their synergistic and cumulative character.

Young people who have a parent with cancer are at risk of ongoing psychosocial problems and previous research has highlighted these young peoples’ need for counselling support (Patterson et al. 2011). The components of ACT presented in *Truce* promote psychological flexibility, skills to cope with difficult thoughts and feelings, mindfulness skills, identification of values and goals, and committed action to living a rich, full and meaningful life. We anticipate that participation in *Truce* will improve psychological well-being in young people who have a parent with cancer. *Truce* has the potential to mitigate many years of mental health problems by building resilience in young people through the skills learnt to successfully deal with their parent's cancer and other potentially psychologically distressing situations in life. The creation of an evaluated, manualised program for young people who have a parent with cancer is also expected to fill a gap in providing support to these young people during this difficult time.

## Abbreviations

ACT: Acceptance and Commitment Therapy
AFQ-Y: Avoidance and Fusion Questionnaire - Youth
CAMM: Child and Adolescent Mindfulness Measure
FRI: Family Relationship Index
K10: Kessler 10
RADS-2: Reynold’s Anxiety and Depression
OCNI: Offspring Cancer Needs Instrument

## Footnotes

1 CanTeen is an Australian charity that provides psychosocial support to 12 to 24 year olds who have cancer, have a close family with cancer, or have had a close family member die from cancer. For more information see: www.canteen.org.au
